# Low‐intensity aerobic exercise improves cardiac remodelling of adult spontaneously hypertensive rats

**DOI:** 10.1111/jcmm.14530

**Published:** 2019-07-17

**Authors:** Luana U. Pagan, Ricardo L. Damatto, Mariana J. Gomes, Aline R. R. Lima, Marcelo D. M. Cezar, Felipe C. Damatto, David R. A. Reyes, Tulio M. M. Caldonazo, Bertha F. Polegato, Marina P. Okoshi, Katashi Okoshi

**Affiliations:** ^1^ Botucatu Medical School, UNESP Sao Paulo State University Botucatu Brazil; ^2^ Itapeva Social and Agrarian Sciences College, FAIT Itapeva Brazil

**Keywords:** arterial hypertension, metalloproteinase, myocardial fibrosis, TIMP

## Abstract

We evaluated the influence of aerobic training on cardiac remodeling in untreated spontaneously hypertensive rats (SHR). Four experimental groups were used: sedentary (W‐SED, n=27) and trained (WEX, n=31) normotensive Wistar rats, and sedentary (SHR‐SED, n=27) and exercised (SHR‐EX, n=32) hypertensive rats. At 13 months old, trained groups underwent treadmill exercise five days a week for four months. Statistical analysis: ANOVA or Kruskal‐Wallis. Exercised groups had higher physical capacity. Hypertensive groups presented left ventricular (LV) concentric hypertrophy with impaired function. Left atrium diameter, LV posterior wall thickness and relative thickness, and isovolumetric relaxation time were lower in SHR‐EX than SHR‐SED. Interstitial collagen fraction and Type I‐Type III collagen ratio were higher in SHR‐SED than W‐SED. In SHR‐EX these parameters had intermediate values between W‐EX and SHRSED with no differences between either group. Myocardial matrix metalloproteinase‐2 activity, evaluated by zymography, was higher in SHR‐SED than W‐SED and SHR‐EX. TIMP‐2 was higher in hypertensive than normotensive groups. In conclusion, low intensity aerobic exercise reduces left atrium dimension and LV posterior wall thickness, and improves functional capacity, diastolic function, and metalloproteinase‐2 activity in adult SHR.

## 1 INTRODUCTION

Physical exercise plays an important role in attenuating pathological cardiac remodelling.^1^ However, the effects of exercise under persistent arterial hypertension are less understood. Spontaneously, hypertensive rats (SHR) subjected to long‐term voluntary wheel running 2, 3, 4 have presented worse cardiac remodelling than sedentary SHR. In voluntary, wheel running SHR usually perform short series of relatively high‐intensity stints reaching many kilometres a day.^5^ It is therefore possible that a high‐intensity exercise programme produces deleterious cardiac effects when performed under increased afterload. In fact, low‐intensity swimming improved adverse remodelling and myocyte contractility in young SHR.^6^ We also observed that light treadmill exercise over four months reduced myocardial fibrosis and attenuated ventricular dysfunction in ageing SHR.^7^ In this study, we evaluated the influence of physical training performed before heart failure development on cardiac remodelling in adult SHR. As the effects of physical exercise on extracellular matrix changes during cardiac remodelling have been poorly addressed, our focus was on myocardial collagen tissue.

## 2 METHODS

The study protocol was approved by Botucatu Medical School Ethics Committee. Twelve‐month‐old male rats were divided into four groups: sedentary Wistar (W‐SED, n = 27); exercised Wistar (W‐EX, n = 31); sedentary SHR (SHR‐SED, n = 27); and exercised SHR (SHR‐EX, n = 32). Training protocol was started at 13 months of age and maintained for 16 weeks. We performed echocardiogram and assessed maximum functional capacity and blood pressure in non‐anesthetized rats before and at the end of the exercise.

Maximum exercise capacity was assessed on a graded treadmill as previously described.^8^ Exercise protocol consisted of 45 min/day treadmill running 5 days/week.^9^ The average treadmill velocity was 17 m/min.

### 2.1 Echocardiography

Echocardiogram was performed under anaesthesia as previously described.^10^, ^11^

### 2.2 Histology

Myocyte diameters were measured as the shortest distance between borders drawn across the nucleus.^12^ Sirius Red F3BA‐stained slides were used to quantify interstitial collagen fraction.^13^

### 2.3 Myocardial hydroxyproline

Hydroxyproline (HOP) concentration was assessed to estimate tissue collagen content (QuickZyme Hydroxyproline Assay, Leiden, Netherlands).

### 2.4 Western blotting

Protein levels were analysed by Western blotting 14 using antibodies Anti‐Type I collagen (sc‐8784‐r; Santa Cruz Biotechnology), Type III collagen (ab6310; Abcam), lysyl oxidase (ab60178; Abcam), TIMP‐1 (R&D Sistems, 150906), TIMP‐2 (Novus Biologicals, NB100‐92000), and GAPDH.

### 2.5 Zymography

Zymography of metalloproteinase (MMP)‐2 was performed as previously described.^7^

### 2.6 Statistical analysis

Results are expressed as mean and standard deviation or median and percentiles. Variables were compared by analysis of variance (ANOVA) for a 2 × 2 factorial design followed by Tukey's test or Dunn's test. Statistical significance: *P* < 0.05.

## 3 RESULTS

Blood pressure was higher in hypertensive groups than their respective controls and unchanged by exercise. Exercise increased functional capacity in exercised groups compared with sedentary rats (Figure S1).

Echocardiographic data are shown in Tables S1 and S2. Hypertensive groups presented higher LV diastolic diameter‐to‐body weight ratio, left atrium diameter (LA)‐to‐body weight ratio, LV mass index, LV posterior wall thickness, relative wall thickness, Tei index, and isovolumetric relaxation time (IVRT), and lower posterior wall shortening velocity than their controls. SHR‐EX had lower LV posterior wall thickness, LA, relative wall thickness, and IVRT than SHR‐SED. Exercise did not change systolic function.

**Figure 1:**
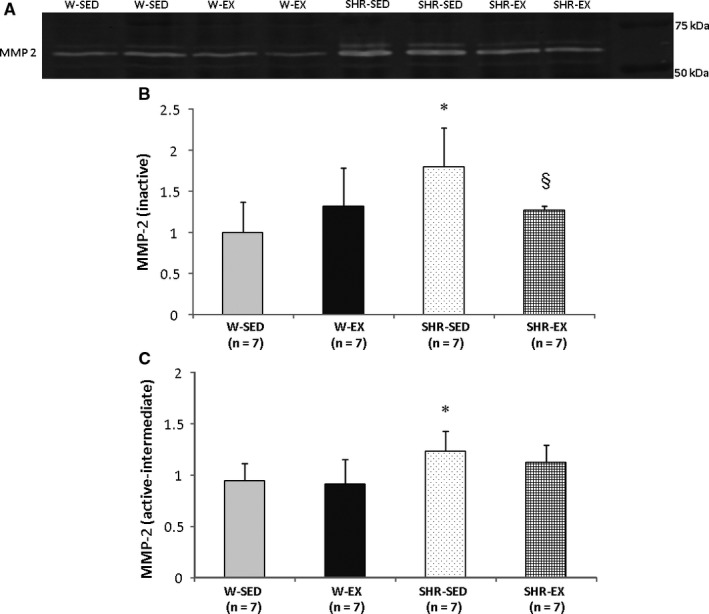
Representative zymography of myocardial matrix metalloproteinase (MMP)‐2 activity (A). Quantification of inactive metalloproteinase‐2 (MMP‐2) activity (B). Quantification of active and intermediate MMP‐2 activity (C). SHR‐EX: exercised SHR; SHR‐SED: sedentary spontaneously hypertensive rats (SHR); W‐EX: exercised Wistar rats; W‐SED: sedentary Wistar rats; Data are mean and standard deviation; ANOVA, and Tukey's; *P < 0.05 vs W‐SED; §*P* < 0.05 vs SHR‐SED

Atrial weight was lower in SHR‐EX than SHR‐SED. Myocyte diameter was statistically larger in hypertensive groups than their controls (W‐SED 16.4 ± 1.11; W‐EX 15.5 ± 1.18; SHR‐SED 17.8 ± 0.96; and SHR‐EX 17.5 ± 0.45 µm). Interstitial collagen fraction (W‐SED 4.26 ± 1.19; W‐EX 5.20 ± 2.11; SHR‐SED 6.49 ± 1.00; and SHR‐EX 6.34 ± 1.92%) was higher in SHR‐SED than W‐SED. Hydroxyproline concentration did not differ between groups. Protein expression is shown in Table 1. Results from zymography of MMP‐2 are shown in Figure 1.

**Table 1:** Protein expression

	W‐SED	W‐EX	SHR‐SED	SHR‐EX
Collagen (Col) I	1.00 ± 0.22	1.05 ± 0.10	1.45 ± 0.56	1.57 ± 0.64#
Col III	1.00 ± 0.31	0.83 ± 0.15	0.82 ± 0.24	0.99 ± 0.24
Col I/Col III	1.00 ± 0.27	1.29 ± 0.33	1.79 ± 0.40*	1.59 ± 0.53
Lysyl oxidase	1.00 ± 0.68	0.49 ± 0.15	1.10 ± 0.52	0.86 ± 0.39
TIMP‐1	1.00 ± 0.28	1.03 ± 0.43	0.75 ± 0.45	0.70 ± 0.28
TIMP‐2	1.00 ± 0.57	0.72 ± 0.25	3.68 ± 2.25*	1.42 ± 0.67#

Abbreviations: SHR‐EX: exercised SHR; SHR‐SED: sedentary spontaneously hypertensive rats (SHR); TIMP: tissue inhibitor of metalloproteinases; W‐EX: exercised Wistar rats; W‐SED: sedentary Wistar rats.

Data as mean ± SD.

ANOVA and Tukey's.

* *P* < 0.05 vs W‐SED,

^#^ *P* < 0.05 vs W‐EX.

## 4 DISCUSSION

We applied a low‐intensity aerobic exercise protocol, previously shown to improve cardiac remodelling in ageing SHR.^7^ Final exercise test showed that the protocol was efficient in improving physical capacity. The fact that exercise did not change blood pressure is in accordance with previous studies in aged SHR 2, 7 and suggests that uncontrolled long‐term hypertension is not modulated by physical exercise.

Echocardiogram performed prior to exercise (data not shown) ensured a uniform assignment of rats between groups. As expected, a decrease in structural parameters observed in SHR‐EX was combined with improved diastolic function, characterized by a lower isovolumetric relaxation time in SHR‐EX than SHR‐SED.

Myocardial collagen fibres form a network regulating force transmission during myocyte shortening and resistance to pathological deformation.^15^ Type I collagen is formed of thick fibres with a high tensile strength, and Type III collagen is composed of fibres of small diameter with a lower tensile strength. The amount of collagen and the integrity of the extracellular matrix are modulated by MMPs and TIMPs. MMPS degrade components of the extracellular matrix and co‐ordinate tissue repair to normal and pathological growth. Excessive activation of MMP, especially MMP‐2 and MMP‐9, plays a key role in cardiac remodelling. TIMPs modulate MMPs by blocking their catalytic site.^15^

Interstitial collagen fraction and Type I‐Type III collagen ratio were higher in SHR‐SED than W‐SED. In SHR‐EX, these parameters had intermediate values between W‐EX and SHR‐SED with no significant differences with these groups, suggesting that exercise reduced the amount of collagen and attenuated change in the Type I‐Type III collagen ratio. Activity of inactive MMP‐2 was lower in SHR‐EX than SHR‐SED; TIMP‐2 expression was increased in both SHR‐SED and SHR‐EX compared with controls. Alterations in variables related to the extracellular matrix in SHR‐SED may be associated with changed collagen tissue, which may affect myocardial stiffness and contribute to LV dysfunction. The fact that SHR‐EX did not present these changes may have contributed to cardiac structural and functional improvement.

Studies on the effects of exercise in cardiac remodelling of hypertensive rats have produced disparate results. When subjected to voluntary wheel running, SHR had increased mortality and myocardial fibrosis with impaired LV dilatation, hypertrophy, and diastolic dysfunction.^2^, ^3^, ^4^ On the other hand, young 6 and old 7 SHR subjected to low‐intensity exercise showed improved cardiac remodelling and contractility with reduced fibrosis. Our study shows for the first time that light exercise is safe, reduces LA size, LV posterior wall thickness, and concentric cardiac remodelling, and improves functional capacity, diastolic function, and metalloproteinase‐2 levels in adult SHR. Therefore, data from this study and literature allow us to conclude that the intensity of physical training has a direct influence on adaptations occurring in cardiac geometry and function of untreated hypertensive rats.

## CONFLICT OF INTEREST

**A**uthors report no conflict of interest.

## AUTHORS CONTRIBUTION

LUP and KO contributed to study design, manuscript writing, and fundraising; RLD, MJG, ARRL, MDMC, FCD, DRAR, BFP, TMMC, and MPO contributed to data collection. All authors have approved the final manuscript.

## Data Availability

All data generated or analysed during this study are included in this published article.
