# Supplementary figures and images for: Low‐intensity aerobic exercise improves cardiac remodelling of adult spontaneously hypertensive rats
Source: J Cell Mol Med. 2019 Jul 17;23(9):6504–7. doi: 10.1111/jcmm.14530 (PMC6714166; doi:10.1111/jcmm.14530)

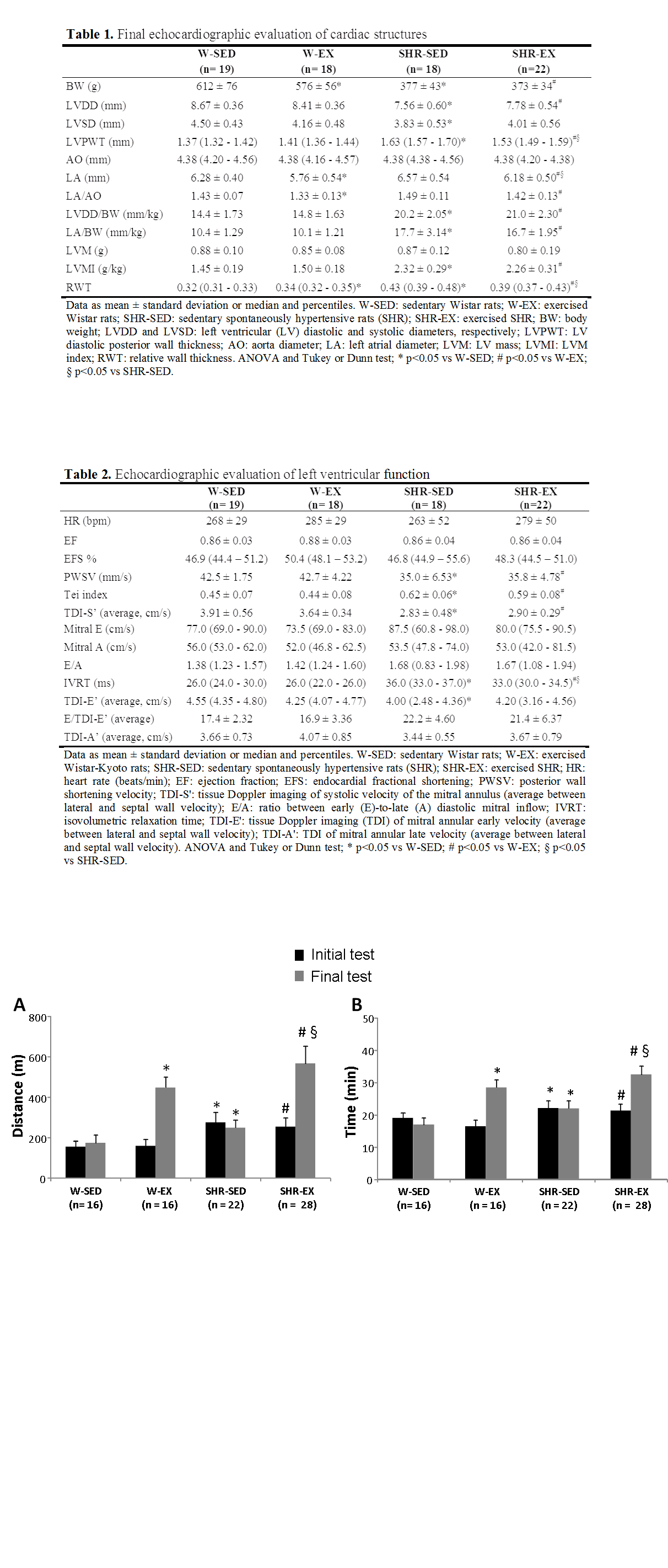

Supplement: Supplementary file 1 [file JCMM-23-6504-s001.tif]
